# Employment of Creosote for the Cure of Affections of the Eye

**Published:** 1841-11

**Authors:** G. T. Black


*Employment of Creosote for the cure of affections of the Eye.* By G. T. Black.—Two years since I suffered severely from inflammation of the eye, occasioned by a cold, and which, perhaps, from inattention, assumed a chronic form. Frequent attempts at removal were made; first by refrigerant and sedative collyria; then by astringents, zinc, nitrate of silver, &c., which produced but slight benefit. Accident, however, shortly did all that I desired—effected the cure.

Whilst replacing on the shelf a bottle of creosote, a small portion of that which usually lodges round the stopper dropped into the affected eye. For a few minutes the pain was extreme; the existing inflammation was also superadded to: these effects were only temporary, soon subsiding into ease, and a sensation of coldness in the eye was experienced. From this time the inflammatory action greatly weakened, till complete restoration took place; subsequently I reflected upon the palpable and sudden alteration for the better, and determined to put the means to the test of experiment, to confirm or annul the claim of the agent. The comparatively few cases, however, which have since fallen under my notice, or rather under my immediate care, do not justify my putting the remedy forward as a specific; yet, in the majority of ophthalmic cases, particularly when of a chronic nature, I believe none of the means now usually employed promise fairer, as a therapeutical agent, for their speedy removal. Three cases are enclosed, in addition to my own, in which I have been happy enough to succeed.

*Case* 1.—A man, aged 40, temperament nervo-bilious; symptoms much as usual, but severe; redness of the conjunctiva adnata, &c.; excessive pain, intolerance of light, and lachrymation; thirst; headach; preternatural heat of body; great pulsation of the temporal arteries, and other febrile symptoms:—venesection to twenty ounces; calomel jalap purges, abstinence, and poppy decoction. These but ill succeeded. Sedative lotions, tartar emetic in nauseating doses—slight benefit. Calomel and opium three times a day: vessels of the conjunctiva still distended, but less bright; general health better; less headach, &c. Patient thoughtlessly absented himself for two days: on next appearance the eyes apparently much the same, but complains of dimness of sight; stimulating lotion; no evident change. Creosote lotion,* to be

*The formula of the lotion employed, gradually increasing the strength according to circumstances, is

R. Creosote, m. iij;
Compound tincture of lavender, m. xx;
Distilled water, ℥ss.

used three times a day. At the expiration of a week, less redness, and no dimness of sight. I have once seen him—quite recovered.

*Case* 2.—A young man, aged 18, (lymphatic temperament,) while at his usual employment had a piece of lime accidentally thrown into his eye, which produced inflammation, that ended in an ulcer near the internal canthus. Upon a subsequent attack from exposure to cold winds the cicatrix spread to a great extent, bordering on the margin of the pupil, attended with indications of chronic inflammation. As far as I conveniently could, the excrescence was removed by knife; the after-application of creosote, in an undiluted state, diminished the remaining portion, and dissipated the inflammation.

*Case* 3.—A child with *ophthalmia tarsi.* It yielded to none of the usually employed remedies, but gave way to the application of the creosote ointment, and the internal administration of the disulphate of quinine. I am fully aware how generally the therapeutical properties of creosote have been called into notice, such as an application to burns and ulcers, in herpetic, furfuraceous, squamous, and crustaceous skin affections, in bronchitis to promote expectoration; in toothach; in atonic rheumatism, &c.; but I have not yet seen any account of its use in affections of the eye, which will, perhaps, excuse me, should such have been the case, for thus taking attention from more important matters.—*Lancet*; *Med. Examiner.*

